# NLP-12/Cholecystokinin signaling stabilizes sensory dendritic structure and protects neuronal healthspan in *Caenorhabditis elegans*

**DOI:** 10.64898/2026.03.05.709874

**Published:** 2026-03-27

**Authors:** Meera M Krishna, Swapnil G Waghmare, Emily C Maccoux, Tania Shaik, Lezi E

**Affiliations:** 1 Department of Cell Biology, Neurobiology and Anatomy, Medical College of Wisconsin, 8701 W Watertown Plank Road, Milwaukee, WI 53226, United States; 2 Neuroscience Research Center, Medical College of Wisconsin, 8701 W Watertown Plank Road, Milwaukee, WI 53226, United States

**Keywords:** aging, neuronal aging, cholecystokinin, neuropeptide, dendrites, PVD

## Abstract

Aging selectively degrades neuronal structure and function, yet the signals that actively preserve neuronal integrity over adult life remain incompletely defined. In *Caenorhabditis elegans*, the PVD sensory neuron develops progressive excessive higher-order dendritic branching during normal aging that correlates with declines in proprioceptive locomotion. Using this system as a quantitative *in vivo* readout of neuronal healthspan, we identify the cholecystokinin-like neuropeptide NLP-12 as a protective signal that preserves PVD homeostasis across adulthood. *nlp-12* loss-of-function animals show early-onset excessive branching and earlier declines in proprioceptive function, whereas *nlp-12* overexpression reduces excessive branching in aged adults without extending lifespan, indicating a neuron-focused effect on healthspan. Using an NLP-12::mKate reporter and coelomocyte uptake as an *in vivo* proxy for secretion, we find that aging is associated with reduced extracellular delivery of NLP-12 and increased retention within the soma of the DVA interneuron, where *nlp-12* is predominantly expressed. Consistent with a requirement for secretory trafficking, disrupting the NLP-12 signal peptide abolishes the rescue effects of *nlp-12* reintroduction in *nlp-12* mutants. Additionally, histamine-gated silencing of DVA during adulthood similarly accelerates PVD excessive branching, supporting an ongoing, adult-stage requirement for this pathway. Receptor genetics further show that the *ckr-1*/GPCR is required for *nlp-12* overexpression-mediated neuroprotection in aged animals. Finally, human cholecystokinin can rescue the branching phenotype in *nlp-12* mutants, supporting evolutionary conservation. Together, these findings implicate conserved cholecystokinin-like neuropeptide signaling as an adult maintenance mechanism that buffers age-associated decline in neuronal resilience.

## 1. INTRODUCTION

Neuropeptides provide an evolutionarily conserved mode of neuromodulatory signaling that can reshape circuit dynamics and behavioral output (Taghert and Nitabach 2012; van den Pol 2012). Unlike fast synaptic transmission, neuropeptides can be released from non-synaptic compartments and engage G-protein coupled receptors (GPCRs) to influence broader cellular neighborhoods. For example, in mammalian hypothalamic oxytocin neurons, neuropeptide release can occur from dendrites and be regulated independently from axon terminal secretion, demonstrating how neuropeptide signaling can act as a distributed communication mode (Ludwig et al. 2002). Neuropeptide systems also play causal roles in behavioral state regulation, as shown by classic manipulations that shift feeding and sleep-wake stability when specific peptidergic pathways are perturbed (Chemelli et al. 1999; Stanley and Leibowitz 1985).

While neuropeptide signaling can influence adult neuronal structure and function, it remains incompletely understood whether peptidergic neuromodulation broadly acts as a long-timescale maintenance program that preserves neuronal integrity as organisms age. This question is especially relevant because neuronal decline is often selective during aging. In line with this principle, distinct neuronal populations show different trajectories of vulnerability across age-related neurodegenerative diseases, suggesting that resilience could be shaped by cell-type-specific properties and by the signals neurons receive from their circuit and tissue environment (Fu et al. 2018; Kampmann 2024; Saxena and Caroni 2011). Neuropeptides are plausible contributors to such differential resilience because GPCR expression is highly cell-type specific (Garcia et al. 2015; van den Pol 2012), such that the same peptide signal can act more strongly in some neurons than others and thereby shape resilience trajectories over time.

To test whether defined neuron-derived peptide pathways can contribute to adult neuronal maintenance in a gene-to-phenotype framework, *Caenorhabditis elegans* (*C. elegans*) can be an ideal model, where neuromodulatory genetics can be directly linked to quantitative phenotypes *in vivo*. Importantly, *C. elegans* contains a large and diverse neuropeptide repertoire and conserved peptide biogenesis machinery. Genome-scale analyses identified extensive neuropeptide-like protein (*nlp*) gene families, indicating broad peptidergic signaling capacity (Nathoo et al. 2001). *C. elegans* also uses conserved neuropeptide biogenesis and processing machinery, in which proprotein convertases such as EGL-3 cleave proneuropeptides and carboxypeptidases such as EGL-21 remove C-terminal basic residues to generate mature bioactive peptides(Jacob and Kaplan 2003).

One conserved neuropeptide family represented in this repertoire is cholecystokinin (CCK), whose receptor signaling is a canonical example of GPCR-based neuromodulation with broad physiological impact across animals. In *C. elegans*, *nlp-12* encodes a CCK-like neuropeptide that is expressed prominently in the single interneuron DVA, a stretch-sensitive proprioceptor that modulates locomotion (Bhattacharya et al. 2014; Chen et al. 2024; Li et al. 2006). NLP-12 has been shown to signal through the GPCRs CKR-1 and CKR-2, which are homologs of mammalian cholecystokinin receptors, and prior work showed that this pathway modulates state-dependent locomotor and foraging behaviors through defined receptor-dependent mechanisms (Bhattacharya et al. 2014; Chen et al. 2024; Hu et al. 2011; Hu et al. 2015; Ramachandran et al. 2021). These ligand-receptor relationships provide a genetic framework for linking conserved neuropeptide signaling to *in vivo* adult phenotypes.

In *C. elegans*, the PVD sensory neuron provides a quantitative structural readout of neuronal aging. During normal aging, PVD neurons progressively develop excessive higher-order dendritic branching that correlates with age-related proprioceptive decline (Krishna et al. 2025). This phenotype reflects active remodeling rather than static overgrowth. Higher-order branches are enriched with F-actin and remain highly dynamic in *vivo*, with ongoing extension and retraction and a net bias toward excessive outgrowth in aged animals (Krishna et al. 2025). Importantly, PVD aging phenotype can be shifted by non-cell-autonomous signals, including epidermal antimicrobial peptide signaling and extracellular matrix remodeling (E et al. 2018; Krishna et al. 2025), providing a useful context to identify additional pathways that modulate neuronal health during adulthood.

Here we show that NLP-12 functions as a protective neuromodulatory signal that promotes PVD dendrite homeostasis during adulthood. Loss-of-function mutation in *nlp-12* induces early-onset excessive higher-order dendritic branching and proprioceptive decline, while *nlp-12* overexpression mitigates the severity of excessive branching in aged animals without affecting lifespan. Our results further indicate that secretion of NLP-12 from DVA is required for this neuroprotective effect during aging-associated remodeling. We also identify CKR-1 as a key component required for this protection, linking a defined peptide-receptor pathway to an adult-onset structural aging phenotype *in vivo*. Expression of human CCK can rescue the early-onset aging phenotype in *nlp-12* mutants, supporting evolutionary conservation. Together, these findings reveal that conserved CCK-like neuropeptide signaling can support neuronal structural resilience during normal aging.

## 2. RESULTS

### 2.1 Loss-of-function mutation of *nlp-12* triggers early-onset aging-associated PVD excessive dendritic branching and proprioceptive deficits

**Figure 1:**
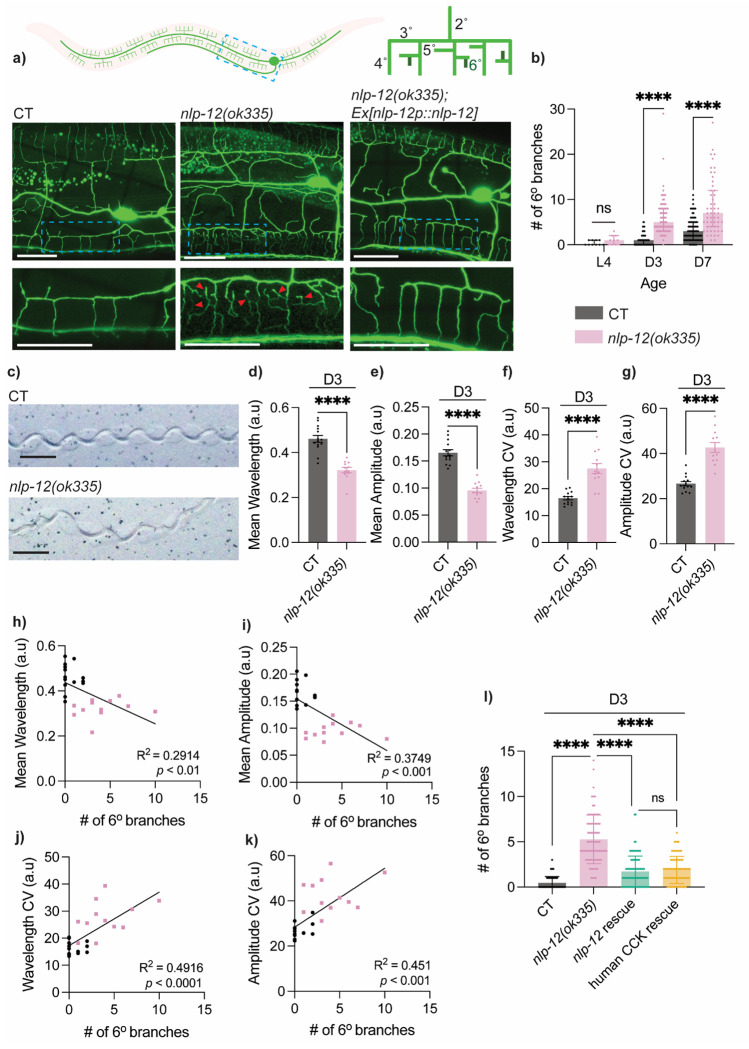
Loss of *nlp-12* triggers early-onset structural and functional aging of PVD neuron **a)** Top left: Illustration of PVD mechanosensory neuron with a blue box indicating region imaged. Top right: Illustration of a PVD dendritic menorah unit with dark green indicating 6° dendritic branches. Representative images of control (CT) animals [*F49H12.4::GFP(wdIs51)]*, *nlp-12(ok335)* mutants, and rescue in *nlp-12* mutant (driven by *nlp-12p*, line 1) animals at Day 3 of adulthood (D3). Red arrowheads indicate representative 6° dendritic branches. Scale bar = 25 μm. **b)** Quantification of number of 6° dendrites in CT and *nlp-12* mutants during aging (L4 CT: *n* = 10, L4 *nlp*-12 mutant: *n* = 10, D3 CT: *n* = 80, D3 *nlp-12* mutant: *n* = 75, D7 CT: *n* = 82, D7 *nlp-12* mutant: *n* = 53). **c)** Representative tracks from CT and *nlp*-12 mutants at D3. Scale bar = 500 μm **d-e)** Mean wavelength *(d)* and amplitude *(e)* measured from 100 tracks per animal and normalized to body length (CT: *n* = 14, *nlp-12* mutant: *n* = 12). **f-g**) Within-individual variability in wavelength *(f)* and amplitude *(g)* measured via coefficient of variability (CV) from 100 tracks per animal (CT: *n* = 14, *nlp-12* mutant: *n* = 12). **h-k)** Correlation between proprioception measurements and number of 6° branches at D3. Each data point corresponding to an individual animal. Proprioception experiments performed without FUDR. Black dots indicates CT, and light pink dots indicate *nlp-12(ok335)*. **l)** Quantification of number of 6° dendrites in CT (*n* = 76), *nlp-12* mutants (*n* = 81), *nlp-12p::nlp-12* rescue in *nlp-12(ok335)* (line 1) (*n* = 86), and human cholecystokinin (CCK) rescue in *nlp-12(ok335)* (driven by *nlp-12p* with *nlp-12* signal peptide, line 1) (*n* = 79) at D3. Unpaired two-tailed *t*-tests were used for two group comparisons, and Kruskal–Wallis test with Dunn’s correction was used for multiple group comparison. Pearson’s correlation test was applied for correlation analyses. ns—not significant, **** *p* <0.0001.

The PVD neurons are a pair of bilateral mechanosensory neurons composed of an extensive dendritic arbor and a singular axon that synapses onto a few interneurons in the ventral nerve cord (Smith et al. 2010; White et al. 1986) (Fig. 1a). From the PVD soma, two primary (1°) dendritic branches extend anteriorly and posteriorly. Secondary (2°) dendrites emerge orthogonally from 1° branches at regular intervals, forming non-overlapping ‘menorah-like’ repeating units that are mainly composed of tertiary (3°) and quaternary (4°) dendrites (Fig. 1a) (Smith et al. 2010). As a polymodal neuron, PVD senses multiple stimuli, including harsh touch, proprioception and cold temperature (Chatzigeorgiou et al. 2010; Tao et al. 2019). Previous work from our lab identified excessive dendritic branching as a robust, quantifiable hallmark of PVD neuronal aging and demonstrated that its severity is tightly associated with aging-associated proprioceptive decline (Krishna et al. 2025). This excessive branching manifests as increasing numbers of 5° and 6° “higher-order” branches in aged animals, with a progressive increase over the course of normal aging (Krishna et al. 2025) (Fig. 1a–b).

Given that this is a progressive, age-dependent phenotype, we asked whether neuromodulatory signals that can act over long timescales contribute to maintaining PVD dendritic homeostasis during adulthood. We focused on the conserved CCK-like neuropeptide *nlp-12*, which is produced prominently by the proprioceptive interneuron DVA and signals through the GPCRs CKR-1 and CKR-2 (Bhattacharya et al. 2014; Chen et al. 2024; Hu et al. 2011; Hu et al. 2015; Li et al. 2006; Ramachandran et al. 2021). We found that animals carrying a loss-of-function mutation in *nlp-12*(*ok335*) exhibited early-onset PVD excessive dendritic branching at Day 3 of adulthood (D3), a young adult time point. Excessive branching was quantified by the number of the aforementioned 6° “higher-order” branches (Fig. 1a–b). Interestingly, by visual inspection, in *nlp-12* mutants we did not detect an obvious change in age-associated dendritic beading, which is another well-established aging-related phenotype of PVD (E et al. 2018), suggesting specificity in the effects of losing *nlp-12*.

We next examined proprioceptive performance in *nlp-12* mutants and observed significant deficits at D3 (Fig. 1c–g). Proprioception, the integration of sensorimotor cues that supports posture and coordinated movement, can be assessed in *C. elegans* by quantifying the track patterns left by individual worms as they move sinusoidally across bacterial lawns (Fig. 1c). Specifically, *nlp-12* mutants showed reduced mean wavelength and amplitude of locomotor tracks (Fig. 1d–e), consistent with what has been reported by others (Tao et al. 2019). We also found further evidence of proprioceptive disruption, with *nlp-12* mutants displaying increased within-individual variability in both track wavelengths and amplitudes (Fig. 1f–g), a feature strongly associated with aging in wild-type *C. elegans* (Krishna et al. 2025). These within-individual variabilities resemble the gait pattern changes reported in older humans, including decreased stride length and increased step-to-step variability, which are linked to impaired proprioception (Callisaya et al. 2010; Wiesmeier et al. 2015). Consistent with our previous findings in wild-type aging animals (Krishna et al. 2025), here we also observed a strong correlation between excessive dendritic branching and proprioceptive deficits (Fig. 1h–k), supporting the behavioral relevance of the neuronal morphological phenotype.

In order to determine whether these phenotypes reflect aging-related changes rather than developmental defects resulting from the loss of *nlp-12*, we performed a series of control experiments. While *nlp-12(ok335)* animals exhibited a shortened lifespan (Fig. S1a), we did not observe any effect on PVD structure at the L4 stage (last/4^th^ larval stage), including the number of 4° dendritic branches or the number of menorah units (Fig. S1b–c). In addition, because *nlp-12* mutants were modestly longer in body length than wild type, we normalized 6° branch number to individual body length and still observed significantly elevated 6° branching in *nlp-12(ok335)* animals (Fig. S1f–g). Moreover, after normalizing 6° branch number to either 4° branch number or menorah number, the excess 6° branches in *nlp-12(ok335)* animals also remained evident and followed an aging-associated progressive trajectory (Fig. 1b; Fig. S1d–e). Given that PVD neurons are born post-embryonically and complete dendritic arborization around late L4 (Smith et al. 2010), these data support the conclusion that the excessive dendritic branching observed in *nlp-12(ok335)* is specifically aging-associated.

Because PVD proprioceptive signaling involves mechanosensory DEG/ENaC channel components (Tao et al. 2019), we performed an exploratory analysis of *mec-10* and *del-1*, which encode DEG/ENaC subunits implicated in PVD proprioceptive function. At D3, *mec-10* mutants showed no obvious change in PVD 6° branch number compared to wild type (Fig. S1h). In contrast, *del-1* mutants displayed reduced 6° branches while showing similar 4° branch number and increased menorah units (Fig. S1h–j), indicating a structural signature distinct from the selective increase in higher-order branching observed in *nlp-12* mutants. These observations argue that *nlp-12* loss produces a branching phenotype that is not readily explained by general disruption of these mechanosensory components.

### 2.2 Human CCK can substitute for NLP-12 to suppress excessive PVD dendritic branching in *nlp-12* mutants

We next performed rescue experiments by reintroducing wild-type *nlp-12* under its native promoter, which significantly reduced the excessive dendritic branching in mutant animals (Fig. 1a, l; Fig. S1k–l), confirming that the branching phenotype in mutant animals is attributable to loss of *nlp-12.* NLP-12 has been proposed as a functional homolog of mammalian cholecystokinin (CCK), based on its sequence similarity to CCK-family peptides and CCK-receptor-family GPCR signaling logic in *C. elegans* (Bhattacharya et al. 2014). To directly test its functional conservation in our PVD aging phenotype, we asked whether human CCK could substitute for NLP-12 *in vivo*. We expressed human CCK cDNA under the *nlp-12* promoter together with the *nlp-12* signal sequence to support proper secretion. This human CCK expression significantly reduced excessive dendritic branching in *nlp-12(ok335)*, to a similar extent as *C. elegans nlp-12* rescue (Fig. 1l; Fig. S1l). Taken together, these findings support functional conservation of CCK-like neuropeptide signaling and emphasize the translational relevance of NLP-12-regulated pathways in preserving neuronal integrity during aging.

### 2.3 Aging is associated with reduced NLP-12 secretion, and secretion is required for NLP-12 regulation of PVD dendritic branching

**Figure 2:**
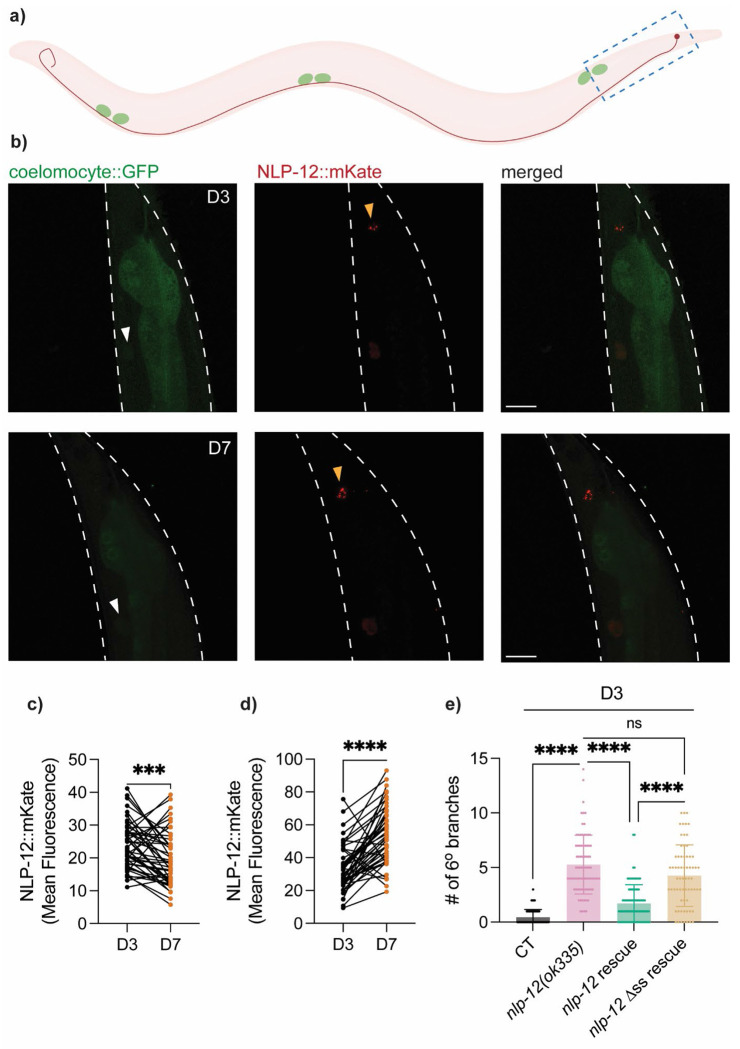
Secretion of NLP-12 is critical for its role in regulating PVD dendritic branching **a)** Illustration of DVA neuron and coelomocytes. Three pairs of coelomocytes indicated in green, and DVA soma and neurite indicated in red. Blue dashed box indicates region shown in representative confocal images in *b)*. **b)** Representative images of coelomocytes and DVA interneuron in D3 and D7 CT animals *[unc-122p::GFP, nlp-12p::nlp-12::mKate]*. *unc-122p::GFP* was used to visualize coelomocytes. White arrowheads indicate coelomocytes. Yellow arrowheads indicate DVA soma location. White dashes indicate outline of an animal. Scale bar = 25μm. **c-d)** Quantification of mean NLP-12::mKate fluorescence averaged across all coelomocytes *(c)* and within DVA soma *(d)* in the same animals at D3 and D7 (*n* = 48). **e)** Quantification of number of 6° dendrites in CT (*n* = 76), *nlp-12(ok335)* (*n* = 81), *nlp-12p::nlp-12* rescue in *nlp-12(ok335)* (line 1) (*n* = 86), and *nlp-12p::nlp*-12 ΔSS (mutated signal sequence) rescue in *nlp-12* mutant (driven by *nlp-12p*, line 1) (*n* = 66) at D3. Additional lines in Figure S1. Wilcoxon matched-pairs signed rank test was used for paired comparison, and Kruskal–Wallis test with Dunn’s correction was used for multiple group comparison. ns—not significant, **** *p* <0.0001. *** *p* <0.001.

While we did not observe obvious aging-related alterations in *nlp-12* expression in wild-type animals (Fig. S2a), neuropeptide signaling depends not only on production but also on appropriate secretion. We therefore asked whether NLP-12 secretion from DVA neurons changes with age and whether secretion is required for NLP-12-mediated protection against aging-related changes in PVD dendritic integrity. To monitor NLP-12 secretion *in vivo*, we first constructed a translational reporter expressing NLP-12::mKate under the *nlp-12* promoter. We simultaneously labeled coelomocytes, six scavenger cells in *C. elegans* positioned near the head, mid-body, and tail that continuously endocytose pseudocoelomic fluid and internalize secreted proteins (Fares and Greenwald 2001) (Fig. 2a). Leveraging this system, we can examine NLP-12::mKate fluorescence in coelomocytes as a proxy for secreted NLP-12 levels, while fluorescence associated with the DVA soma region provides an estimate of intracellular retention. Additionally, to ensure a more consistent analysis, we imaged the same animals at D3 and again at D7 and quantified NLP-12::mKate signal in both compartments at each time point, allowing within-animal comparisons of NLP-12 levels across age. With this, we observed reduced NLP-12::mKate accumulation in coelomocytes with age, accompanied by increased signal at the DVA soma region (Fig. 2b–d), suggesting an age-associated reduction in NLP-12 secretion.

To test the importance of secretion in NLP-12’s function in limiting PVD excessive branching, we engineered a secretion-defective *nlp-12* rescue construct by mutating the signal peptide, substituting the first four amino acids of the hydrophobic core with hydrophilic glutamic acids. Disrupting the hydrophobic core is expected to impair signal peptide function and thereby reduce entry of NLP-12 into the secretory pathway. In contrast to the wild-type *nlp-12* rescue construct, expression of this signal peptide mutant construct failed to reduce the excessive PVD dendritic branching phenotype of *nlp-12(ok335)* animals (Fig. 2e, Fig. S2b). Together, these findings indicate that NLP-12 secretion is required for its protective effect on PVD dendritic structure and suggest that an aging-associated reduction in NLP-12 secretion could contribute to the aging-associated excessive dendritic branching of the PVD neuron.

### 2.4 Overexpression of *nlp-12* attenuates aging-associated PVD excessive dendritic branching without altering lifespan

**Figure 3:**
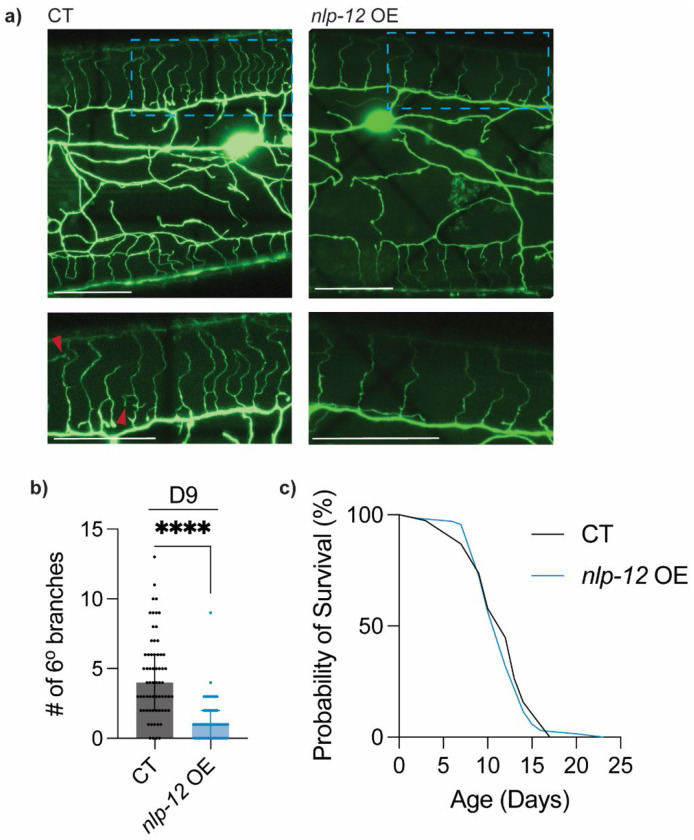
Overexpression of *nlp-12* attenuates aging-associated excessive dendritic branching **a)** Representative images of CT and *nlp-12* overexpression (OE) driven by *nlp-12p* (line 1) at D9.Red arrowheads indicate 6° branches. Scale bar = 25μm. **b)** Quantification of number of 6° dendrites in CT (*n* = 65) and *nlp-12* OE driven by *nlp-12p* (line 1) (*n* = 63) at D9. **c)** Lifespan comparison of CT and *nlp-12* OE driven by *nlp-12p* (line 1) (Starting *n* = 100). No statistically significant difference between CT and OE animals. Additional replicates and lines in Figure S3. Lifespan experiment performed without FUDR. Mann–Whitney test was used for two group comparison and survival data was evaluated using Kaplan–Meier analysis with log-rank (Mantel–Cox) tests. **** *p* < 0.0001.

Given the importance of NLP-12 secretion in this context, we next asked whether increasing NLP-12 availability is sufficient to counteract aging-associated PVD excessive branching. Transgenic overexpression of *nlp-12* driven by its native promoter in wild-type animals significantly reduced excessive dendritic branching at D9 when compared to control animals (Fig. 3a–b, Fig. S3a). Notably, this overexpression of *nlp-12* did not alter lifespan, indicating that enhanced NLP-12 signaling can specifically improve this neuronal aging process without broadly affecting systemic aging (Fig. 3c, Fig. S3b). Together with our finding that human CCK can substitute for NLP-12 in suppressing PVD excessive branching (Fig. 1l), these data support the idea that this conserved CCK-like neuropeptide signaling can modulate neuronal structural aging in a manner that is separable from lifespan regulation.

### 2.5 Silencing the NLP-12-producing DVA interneuron triggers early-onset PVD excessive dendritic branching

**Figure 4:**
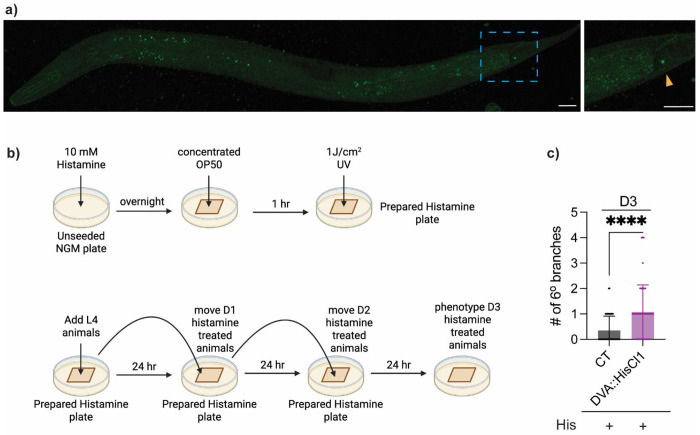
Silencing the NLP-12-producing DVA interneuron triggers early-onset PVD excessive dendritic branching **a)** Representative image of an L4 stage animal with endogenous *nlp-12* locus tagged with T2A::3xNLS::GFP (*syb3112*[*nlp-12*::T2A::3×NLS::GFP]) demonstrating DVA neuron specific expression of *nlp-12*. Yellow arrowhead indicates location of DVA soma. Scale bar = 25μm. Mild autofluorescence of gut granules, a normal physiological feature in *C. elegans*, appears as small punctate structures throughout the body. **b)** Schematic illustrating histamine plate preparation and treatment protocol. Created with Biorender. **c)** Quantification of number of 6° branches in CT (*n* = 46) and DVA::HisCl1 (*nlp-12p* driven expression of *Drosophila* HisCl1 channel, line 1) (*n* = 45) at D3. + indicates presence of histamine (His) on plates from L4 to D3 stage. Experiments performed without FUDR. Mann-Whitney test was used for two group comparison. **** *p* < 0.0001.

Since NLP-12 is produced predominantly by the DVA interneuron (Bhattacharya et al. 2014; Chen et al. 2024; Li et al. 2006), we next asked whether normal DVA activity is required to restrain aging-associated PVD excessive branching. We first confirmed the expression pattern of *nlp-12* using an endogenous *nlp-12* locus tagged with T2A::3xNLS::GFP, which showed signal restricted to the DVA soma (Fig. 4a).

To selectively silence DVA, we generated a transgenic strain expressing the *Drosophila* histamine-gated chloride channel HisCl1 under the *nlp-12* promoter, which has been widely used to label or manipulate the DVA interneuron (Choi et al. 2015; Wen et al. 2012), and treated these animals with exogenous histamine. This chemogenetic approach enables inducible neuronal silencing as histamine opens HisCl1 to drive chloride influx and suppress neuronal activity (Pokala et al. 2014). Because *C. elegans* lacks endogenous histamine-gated chloride channels, histamine has minimal effects in animals and cells lacking HisCl1 and can therefore selectively inhibit neurons that transgenically express the channel (El Mouridi et al. 2021; Pokala et al. 2014). We initiated histamine treatment at L4 stage and continued until D3 (Fig. 4b). Under these conditions, we observed that DVA silencing resulted in early-onset excessive dendritic branching by D3 (Fig. 4c, Fig. S4a), suggesting that the normal DVA activity is required for preserving PVD dendritic integrity during aging.

We next considered whether the *nlp-12* promoter could drive HisCl1 outside DVA in a way that indirectly affects PVD branching. Given that minimal-level *nlp-12* expression has been reported in the pharynx in addition to DVA (Chen et al. 2024), we considered the possibility that *nlp-12p::HisCl1* could also affect pharyngeal activity and thereby alter food intake, which in turn can influence aging-related phenotypes (Lakowski and Hekimi 1998). While we did not directly measure pumping in histamine-treated *nlp-12p::HisCl1* animals, *nlp-12(ok335)* mutants displayed normal pharyngeal pumping under our assay conditions (Fig. S4b), arguing that *nlp-12* does not play a critical role in food intake behavior. In addition, histamine exposure alone did not detectably alter PVD dendritic morphology in animals lacking HisCl1 (Fig. S4c). Together, these controls reduce concern that altered feeding or histamine itself accounts for the early-onset PVD hyperbranching observed upon DVA silencing.

### 2.6 CKR-1/Cholecystokinin receptor functions downstream of NLP-12 to regulate PVD dendritic health during aging

The next step in understanding the mechanism of NLP-12 regulation of PVD neuron aging is to identify potential downstream factors. Because our prior work showed that the apical epidermal collagen *col-120* modulates aging-associated PVD excessive branching in a similar manner (Krishna et al. 2025), we tested its genetic interaction with *nlp-12*. Notably, the severity of excessive branching in *col-120(sy1526)* animals was comparable to that in *nlp-12(ok335)*, and double mutants also did not show enhanced hyperbranching relative to either single mutant (Fig. S5a). Surprisingly, loss of *col-120* eliminated the beneficial effects conferred by *nlp-12* overexpression in aged animals (Fig. S5b). These results suggest that epidermal collagen state can gate the phenotypic impact of increased NLP-12 signaling, but they do not identify the signaling components that directly receive and transmit the NLP-12 cue.

We therefore tested the hypothesis that this neuropeptide signal is transmitted through downstream receptors, as neuropeptide ligands typically exert their effects via specific GPCRs. Prior work has identified CKR-1 and CKR-2, both highly conserved homologs of mammalian cholecystokinin receptors, as functional receptors for NLP-12 in regulating state-dependent locomotor and foraging behaviors (Bhattacharya et al. 2014; Chen et al. 2024; Hu et al. 2011; Hu et al. 2015; Peeters et al. 2012; Ramachandran et al. 2021). In addition, NPR-2 and NPR-5 are two other GPCRs that share sequence homology with CKR-1 and CKR-2 and have been proposed as potential targets for NLP-12 signaling (Bhattacharya et al. 2014).

**Figure 5:**
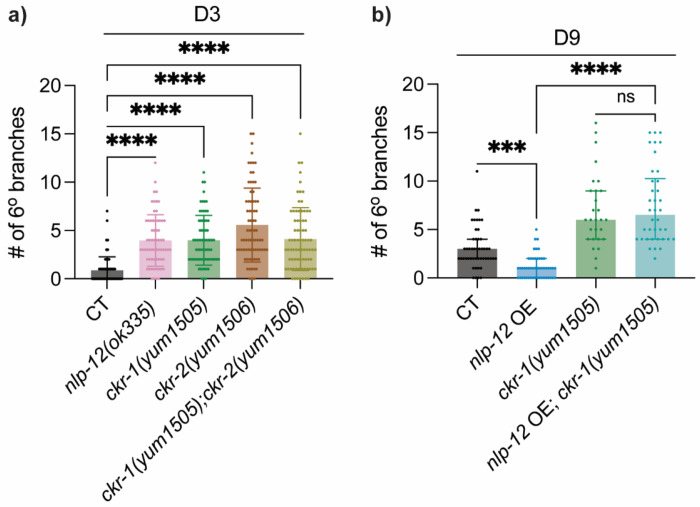
NLP-12 acts via its receptor CKR-1/GPCR to regulate PVD dendritic health during aging **a)** Quantification of number of 6° branches in CT (*n* = 77), *nlp-12(ok335)* mutant (*n* = 65), *ckr-1(yum1505)* mutant (*n* = 71), *ckr-2(yum1506)* mutant (*n* = 75) and *ckr-1(yum1505); ckr-2(yum1506)* double mutant (*n* = 77) at D3. **b)** Quantification of number of 6° branches in CT (*n* = 49), *nlp-12* OE driven by *nlp-12p* (*n* = 50), *ckr-1(yum1505)* mutant (*n* = 28), *nlp-12* OE in *ckr-1(yum1505)* mutant background (*n* = 38) at D9. Kruskal–Wallis test with Dunn’s correction was used for multiple group comparisons ns—not significant, *** *p* < 0.001, **** *p* < 0.0001.

Upon examining mutants for these receptor candidates, we found that both *ckr-1(yum1505)* and *ckr-2(yum1506)* animals exhibit early-onset excessive PVD dendritic branching at D3, to a similar extent as that observed in *nlp-12(ok335)*, while PVD dendritic morphology was unaffected in *npr-2(ok419)* or *npr*-5*(ok1583)* animals (Fig. 5a, Fig. S5c). These results argue that the PVD excessive dendritic branching phenotype is not broadly sensitive to perturbing GPCR signaling, and instead is selectively associated with loss of CKR-1 and CKR-2. Notably, *ckr-1;ckr-2* double mutants demonstrated no enhanced branching compared to the single mutants (Fig. 5a), suggesting that CKR-1 and CKR-2 may act in a shared genetic pathway in this context.

While CKR-1 and CKR-2 are both established NLP-12 receptors, we prioritized CKR-1 as a representative receptor to test whether NLP-12’s protective effects on PVD dendritic integrity during aging requires downstream receptor signaling. We overexpressed *nlp-12* in the *ckr-1(yum1505)* null background and found that the loss of *ckr-1* completely eliminated the beneficial effects from *nlp-12* overexpression on PVD dendrite structure during aging (Fig. 5B). Collectively, these data suggest that NLP-12 preserves PVD dendritic integrity during aging through downstream CCK receptor-like GPCR signaling.

## 3.4 DISCUSSION

Adult neurons must balance plasticity with stability throughout life, yet the signals that actively restrain aging-associated structural and functional changes *in vivo* remain poorly defined. In this study, using the *C. elegans* PVD sensory neuron as a quantitative readout, we identify a conserved CCK-like neuropeptide signaling as an adult maintenance input that buffers an aging-associated dendritic branching remodeling program. Loss of *nlp-12* accelerates excessive dendritic branching and proprioceptive decline that are typically seen in aged wild-type animals, while restoring the peptide signaling, including by human CCK, suppresses premature changes in dendritic structure. Our receptor genetics indicate that CKR-1/GPCR is required for NLP-12-dependent neuroprotection, highlighting that age-associated neuronal decline may be tuned through a receptor-defined neuromodulatory pathway. Collectively, these findings establish a tractable *in vivo* framework for dissecting how neuromodulatory signals are deployed across adulthood to sustain neuronal healthspan, without necessarily altering organismal lifespan.

Prior work defined NLP-12 as a DVA-derived CCK-like neuropeptide that signals through the GPCRs CKR-1 and CKR-2 to shape locomotor and foraging-related behaviors (Bhattacharya et al. 2014; Hums et al. 2016; Janssen et al. 2008; Ramachandran et al. 2021). Importantly, this ligand-receptor axis has been anchored to distinct circuit and effector sites. CKR-2 functions in cholinergic motor neurons to modulate neuromuscular transmission, and CKR-1 has been implicated in head motor circuitry including SMD neuron (Chen et al. 2024; Hu et al. 2011; Hu et al. 2015; Ramachandran et al. 2021). Our work expands this axis into an aging-relevant role by linking NLP-12 signaling to adult neuronal maintenance, connecting a defined ligand-receptor system to both structural and functional aging phenotypes and identifying CKR-1 as a required component for protection in this context. However, this raises the question of which step becomes limiting with age.

Mechanistically, our results highlight peptide availability as an age-sensitive control point for NLP-12 signaling. While *nlp-12* expression does not show a strong age-linked change, aging is accompanied by reduced coelomocyte-associated NLP-12 signal and increased retention in DVA soma, consistent with diminished effective secretion over time. Coelomocyte uptake has been widely used as an *in vivo* proxy for secreted material and has been applied to quantify neuropeptide release using established controls (Ch’ng et al. 2008; Fares and Greenwald 2001; Hoover et al. 2014), although it remains an indirect readout that can be influenced by coelomocyte activity and whole-animal physiology.

Nevertheless, consistent with a secretion-dependent mechanism, the failure of a signal-sequence-mutant *nlp-12* construct to rescue the excessive branching phenotype in *nlp-12* mutants indicates that correct routing through the secretory pathway, and thus extracellular signaling, is required for protection, although signal-sequence disruption may also affect processing or peptide stability. Together, these observations point to potential age-sensitive bottlenecks in dense-core vesicle maturation, cargo sorting or transport, or activity-coupled exocytosis that could reduce peptide output even when expression persists (Laurent et al. 2018; Sasidharan et al. 2012; Topalidou et al. 2016).

If peptide availability becomes limiting with age, the downstream receptors become the key gate for whether remaining signal can still be protective. In our aging readout, loss of either *ckr-1* or *ckr-2* phenocopies *nlp-12* loss, while the *ckr-1;ckr-2* double mutant is not more severe. Although our data do not yet resolve how the two receptors share this protective output, this non-additivity is consistent with several models for dual-receptor peptide signaling. First, the pathway may be thresholded. Maintaining PVD integrity may require peptide signaling above a minimum level, and removing either receptor reduces effective signaling below that level, producing a saturated phenotype that the double mutant cannot intensify. Second, CKR-1 and CKR-2 may provide non-redundant inputs that converge on the same protective outcome. The receptors could act in different cells, or couple to distinct downstream effectors, such that losing either arm is sufficient to collapse net protective signaling and yields a non-additive double-mutant phenotype. This type of dual-receptor logic has precedent in *C. elegans*, where a single neuropeptide, FLP-18, can engage different receptors, NPR-1 and NPR-4, in distinct neurons to shape a single behavioral output (Bhardwaj et al. 2018). A third possibility is context-dependent redundancy. Prior NLP-12 circuit work in locomotor state control and adaptive foraging behaviors indicates that CKR-1 and CKR-2 can serve as alternate GPCR targets, and that apparent redundancy depends strongly on assay context and readout (Ramachandran et al. 2021). Overall, the non-additive genetics suggest that NLP-12-dependent protection is limited by a thresholded or saturable pathway output, such that loss of either receptor is sufficient to disrupt protection in this assay. Determining whether CKR-2 is also required for the overexpression-mediated protection will clarify how CKR-1 and CKR-2 jointly implement NLP-12-dependent neuroprotection in this aging context.

Given that receptor sites for NLP-12 signaling have been mapped largely outside PVD, an important circuit-level question is how DVA-derived NLP-12 influences PVD branching dynamics if the relevant receptor does not act cell-autonomously in PVD. Consistent with this, connectome data indicate only sparse synaptic connectivity between DVA and PVD (Cook et al. 2019), supporting a model in which NLP-12 acts primarily through extrasynaptic or circuit-mediated routes. Available expression and functional mapping show *ckr-1* is predominantly expressed in multiple head neurons, including SMD and AIB, and in a subset of ventral cord cholinergic motor neurons, without obvious expression in PVD (Chen et al. 2024; Ramachandran et al. 2021; Taylor et al. 2021). Similarly, CKR-2 has been shown to function in cholinergic neurons at the neuromuscular junction, and *ckr-2* has likewise not been reported to be expressed in PVD (Hu et al. 2011; Hu et al. 2015; Taylor et al. 2021). Although speculative, this receptor localization supports an indirect route that NLP-12 influences PVD through circuit-level effects on locomotion and muscle activity, thereby altering the mechanical environment experienced by PVD dendrites across adulthood. Higher-order PVD dendritic branches are actin-rich and remain dynamic *in vivo*, so even small shifts in the balance between extension and retraction events could accumulate into net outgrowth over time (Krishna et al. 2025). Because PVD terminal branches run along the body wall at the interface between the hypodermis and body-wall muscle (Liang et al. 2015), changes in motor state, posture, neuromuscular drive, or muscle tone could plausibly alter the frequency and magnitude of body-wall deformation sensed by these dendritic compartments, biasing branching stabilization or elimination. In this view, NLP-12 does not need to signal within PVD itself. Instead, NLP-12 could act through motor circuits to keep movement and muscle output in a range that favors dendritic stability during adulthood. If the relevant output of this circuit-level signaling is mechanical, then properties of the body-wall tissue scaffold that PVD contacts should also influence how strongly this pathway impacts branching.

Consistent with this idea, our prior work identified the apical epidermal collagen *col-120* as a determinant of aging-associated PVD excessive branching, implicating the epidermal extracellular matrix as a component of the local tissue context that shapes PVD remodeling (Krishna et al. 2025). In the current study, the genetic interaction pattern between *col-120* and *nlp-12* is consistent with *col-120* providing a permissive context for *nlp-12*-dependent regulation of branching. One possible explanation is that NLP-12, acting through motor circuits, influences movement and muscle output in ways that affect the mechanical environment experienced by PVD branches, but the impact of these changes depends on intact epidermal ECM structure. Together, these observations suggest a model in which that circuit-level neuromodulation and epidermal ECM state interact to influence PVD branching during aging.

Our data showing functional rescue by human CCK indicate that key features required for protective CCK signaling are conserved across substantial evolutionary distance. It argues that the protective effect depends on conserved properties of the ligand-receptor logic rather than *C. elegans*-specific properties of NLP-12. More broadly, studies with human postmortem brains have reported region-dependent alterations in CCK and receptor measures in Alzheimer’s disease (AD), with some variability across cohorts and assays (Hays and Paul 1982; Lofberg et al. 1996; Mazurek and Beal 1991; Rossor et al. 1981). More recent human biomarker work with the Alzheimer’s Disease Neuroimaging Initiative (ADNI) also reports that cerebrospinal fluid CCK levels are associated with cognitive performance and neuroimaging changes (Plagman et al. 2019). Additionally, studies with AD mouse models further suggest that CCK-linked circuitry and CCKBR, a principal receptor for CCK, can influence disease-relevant synaptic and cognitive phenotypes (Shi et al. 2020; Zhang et al. 2024). These observations link CCK signaling to mammalian neurodegeneration in disease-relevant contexts, but they do not yet establish whether reduced CCK tone is sufficient to drive age-associated neuronal decline. In that light, our results support targeted tests in vertebrate systems of whether CCK signaling contributes to long-timescale neuronal maintenance during aging, and whether altered peptide availability or release, including secretion-dependent changes, contributes to neuronal vulnerability.

In summary, this work identifies conserved CCK-like neuropeptide signaling as a neuron-derived maintenance input that delays aging-associated dendritic remodeling and preserves proprioceptive function in *C. elegans*. It also elevates peptide delivery as a potentially age-sensitive bottleneck, suggesting a route by which aging can weaken neuromodulatory maintenance signals even when gene expression is largely preserved. More broadly, together with prior demonstrations that non-neuronal tissue cues reshape PVD aging (E et al. 2018; Krishna et al. 2025), these findings support a model in which extrinsic signals from multiple origins, including barrier tissues and other neurons, tune selective aspects of neuronal aging.

## 4. MATERIALS AND METHODS

### 4.1 *Caenorhabditis elegans* Strains and Maintenance

*C. elegans* strains were maintained at 20°C, or as otherwise indicated, on Nematode Growth Medium (NGM) plates seeded with *E. coli* OP50. Only hermaphrodites were used for data generating experiments. For age-synchronized progeny, plates with gravid hermaphrodites were bleached to eliminate microbes, and animals were transferred to NGM plates containing 100 μM 5′fluorodeoxyuridine (FUDR) (VWR, 76345–984) at the mid- or late-L4 stage to prevent egg hatching. Day 1 of adulthood (D1) was defined as 24 hours post-L4. Given the potential confounding effects of FUDR on aging(Feldman et al. 2014; Van Raamsdonk and Hekimi 2011), certain experiments were performed without FUDR, moving animals onto fresh plates daily until D5, and every 2 days thereafter. FUDR-free experiments are noted in figure legends. See Table S1 for strain information.

### 4.2 DNA Constructs and Generation of Transgenes

DNA expression constructs were generated using Gateway cloning technology (Thermo Fisher) (Table S2). Primers were acquired from Millipore and Thermo Fisher (Table S3) Transgenic animals were generated by microinjection, with plasmid DNAs used at 1–100 ng/μL, co-injection marker *ttx-3p::GFP* or *ttx-3p::RFP* at 50 ng/μL (Tables S1 and S2). At least two independent transgenic lines were analyzed per construct. The promoters used were *nlp-12p* established as −320 bp from start codon. Human CCK ORF clone was obtained from Applied Biological Materials (#PC115339).

### 4.3 Fluorescent Microscopy

Neurons were visualized using fluorescent reporters and phenotyped on a Zeiss Axio Imager M2 compound microscope using 0.5%–1% M9 dilution of 1-phenoxy-2-propanol (Fisher) for immobilization and mounted on 4% agarose pads in M9 solution. Confocal images were taken on a Leica SP8 confocal microscope with Z-stack (0.5-1 μm/slice) at 63X magnification, using 5μM Levamisole (Millipore Sigma, 31742) for immobilization. Imaging parameters were kept constant across genotypes within each experiment.

### 4.4 Quantification of Neuron Morphological Defects

*F49H12.4::GFP(wdIs51)* (Smith et al. 2010) was used to visualize PVD neurons. Representative images were taken on a Leica SP8 confocal microscope with a 63X objective and Z-stack projections. Excessive dendritic branching was quantified on a Zeiss Axio Imager M2 compound microscope using 4% agar pads with 5uM Levamisole for immobilization, by counting 6° branches, identified within “menorah” structures. 6° branches for each animal were counted for either PVDL or PVDR for either the dorsal or ventral side, depending on which was clearly visible during the imaging session. For *mec-10* and *del-1* mutants, *ser-2(prom3)::myr::GFP + odr-1p::RFP(wyIs592)III* was utilized to visualize PVD neurons due to challenges in crossing *mec-10(tm1552)X* and *del-1(ok150)X* with (*wdIs51)X*. *wyIs592* animals had larger body size than *wdIs51*, likely due to genetic background differences, which may account for the branching number variation at D3 between the two reporter strains.

For each experimental group, a minimum of three replicates of 30 animals, or as indicated otherwise, were quantified alongside a control group, with experimenters blinded to sample identity to minimize bias. For statistical analysis, see below.

### 4.5 Coelomocyte Imaging

Levels of secreted and retained NLP-12 was assessed via imaging on a Leica SP8 confocal microscope using 2% agarose pads with 5uM Levamisole for immobilization. Experimental animals [*unc-122p::GFP(oxIs253); NLP-12::mKate; ttx3p::GFP* (*lxyEx120)*] were imaged at D3, recovered, and imaged again at D7. Images were Z-stack projections captured with a 63X objective in both red and green channels. 3-4 sections were imaged for each worm to capture the 3 coelomocyte pairs, and the DVA neuron soma. NLP-12::mKate fluorescence was quantified in Fiji by measuring the mean mKate fluorescence within the area demarcated by the coelomocyte fluorescence in GFP to estimate NLP-12 within the coelomocytes, and mean mKate fluorescence in the area corresponding to the predicted location of the DVA soma.

### 4.6 Lifespan Assay

Lifespan assays were conducted at 20 °C in the absence of FUDR. L4 animals were transferred to assay plates and scored daily starting from Day 1 of adulthood. Animals were transferred daily until Day 5 of adulthood and then every other day thereafter to minimize confounding from progeny. Lifespan was calculated from the time animals were first placed on the assay plates until they were scored as dead. An animal was considered dead when it failed to respond to gentle prodding with a platinum wire and displayed no pharyngeal pumping. Animals that died due to vulval rupture, internal hatching (bagging), or desiccation (crawling off the agar) were censored from the analysis. Lifespan was calculated from the time animals were first placed on the assay plates until they were scored as dead. Each condition included 90–100 animals across three biological replicates. The PVD::GFP background strain (*wdIs51*) was used as the control for all experiments.

### 4.7 PVD Function Analysis: Proprioception

Proprioceptive sensing specific to PVD neurons was assessed using a locomotion assay as described previously (Tao et al. 2019; Tavernarakis et al. 1997) with some modifications. On the day of assay, animals were transferred to fresh NGM plates seeded with OP50, one animal per plate, and allowed to roam freely for 2–3 h at 20°C, until sufficient tracks were made in the bacterial lawn. The plate was then imaged using a digital camera attached to a Leica S9i stereomicroscope at 1X magnification. A total of 100 randomly selected wavelengths (distance between two adjacent peaks) and amplitudes (distance between peak and wavelength, perpendicular to wavelength) were measured using Fiji. All measurements were used in cases where animals had fewer than 100 measurements, with a minimum of 40 measurements per animal. All measurements were normalized to the body lengths of individual animals and units were converted to millimeters utilizing a reference slide. All animals subjected to proprioception assay were also examined under the Zeiss to quantify PVD dendritic branching.

### 4.8 Histamine-based DVA silencing protocol

A standard histamine treatment protocol (Pokala et al. 2014) was utilized, with 1M histamine solutions prepared by adding 1.85 g of Histamine dihydrochloride (Sigma-Aldrich, H7250-10G) to 10 mL of ultrapure water and filtered. 150 μL of 1M His was added to unseeded 60mm NGM plates (final concentration of 10 mM) and left to rest overnight. Control plates were prepared with equal volume ultrapure water. OP50 was cultured for 18-20 hours in 50 mL of B-Broth and centrifuged, following which supernatant was discarded, and 50 μL of concentrated OP50 was pipetted and spread onto the prepared plates. After 1 hour to dry, the bacteria were UV-killed in a Spectroline Microprocessor-Controlled UV Crosslinker at 1J/cm^2^. UV-killed OP50 was used to prevent bacterial proliferation and histamine-dependent changes in lawn density or bacterial physiology during the multi-day exposure period, which can alter feeding conditions and confound interpretation of histamine-driven HisCl1 silencing. L4 animals were there added to the prepared plates and moved onto fresh plates every 24 hours until phenotyped at D3. Animals expressing the *Drosophila* HisCl1 channel in pharyngeal muscles [*F49H12.4::GFP(wdIs51); myo-2p::HisCl1::SL2::mScarlet::tbb-2 5’UTR(lxyEx134)*] were used as a positive control to confirm the effectiveness of the prepared histamine plates on all days of the protocol, assessed by examining the pharyngeal pumping of treated positive controls.

### 4.9 Pharyngeal Pumping Assay

To assess pharyngeal pumping in *nlp-12* mutants, healthy actively moving worms were chosen and Leica S9i stereomicroscope was used to capture individual video recordings of pharyngeal pumping of each worm using a 2× objective lens with brightfield illumination. As the worms were unrestrained and freely moving, the plates were placed on a transparent plastic sheet and moved to keep the worm in the lens’ field of view. The captured video was viewed on VLC media player at 0.25CX speed and the number of pharyngeal pumps was counted using a handheld counter for a period of 30 seconds. Worms that were harmed while picking (aberrant track waveforms, unmoving worms, injured worms) and worms not on the bacterial lawn were excluded.

### 4.10 RT-qPCR

To quantify the change in expression level of genes in aging, N2 WT animals at D1 and D7 were collected, washed with M9 solution for three 30-min washes, and total RNA was isolated with TRI reagent (Millipore Sigma). To test RNAi efficacy, 10-worm lysis method (Ly et al. 2015) was used to prepare lysates. Briefly, 10 worms were cleaned in ultrapure H_2_O and placed in 1 μL of lysis buffer (0.25 mM EDTA, 5 mM Tris pH 8., 0.5% Triton X-100, 0.5% Tween-20, 1 mg/mL proteinase K). Samples were lysed in a thermal cycler at 65°C for 15 min, 85°C for 1 min, and stored at −80°C for at least 12 h. An additional 1.5 μL of lysis buffer was then added, and the cycle was repeated. Samples were treated with dsDNAse (Thermo Fisher; 0.5 μL each of 10X buffer and dsDNAse, 1.5 μL ultrapure H2O) and incubated at 37°C for 15 min, 55°C for 5 min, and 65°C for 1 min, followed by reverse transcription. Reverse transcription was performed with iScript Reverse Transcription Supermix for RT-qPCR (BioRad). RT-qPCR was performed using designed primers (Table S3) and iTaq Universal SYBR Green (Bio-Rad). *cdc-42* and *act-2* were used as housekeeping controls. Primer efficiency was verified utilizing standard curve method. Bio Rad CFX96 Touch Deep Well Real-time PCR Detection System was used to perform the qPCR and relative mRNA levels were calculated using comparative ΔΔCT method.

### 4.11 Statistical Analysis

All analyses were performed using GraphPad Prism (v10.4.0). Normality was assessed using the Shapiro–Wilk and D’Agostino & Pearson test. Survival data were evaluated using Kaplan–Meier analysis with log-rank (Mantel–Cox) tests. Pearson’s correlation test was applied for correlation analyses. For multiple group comparisons, one-way ANOVA with Tukey’s post hoc test was used for normally distributed datasets, and Kruskal–Wallis tests with Dunn’s correction were used for non-parametric datasets. For two group comparisons, unpaired two-tailed *t*-tests for parametric data or Mann–Whitney tests for non-parametric data were used. Comparison of NLP-12::mKate levels at D3 and D7 were analyzed using the Wilcoxon matched-pairs signed rank test. Significance was defined as *p* < 0.05. Bar graphs display the mean ± standard error of the mean (SEM) for normally distributed data, and the median with interquartile range for non-normally distributed data, unless otherwise specified. Sample sizes and statistical tests are provided in the corresponding figure legends.

## Supplementary Material

1

## Data Availability

Strains and plasmids are available upon request. The authors affirm that all data necessary for confirming the conclusions of the article are present within the article, figures, and supplementary materials.
