# Supplementary material for: NLP-12/Cholecystokinin signaling stabilizes sensory dendritic structure and protects neuronal healthspan in Caenorhabditis elegans
Source: bioRxiv. 2026 Mar 27:2026.03.05.709874. Preprint. [Version 2] doi: 10.64898/2026.03.05.709874 (PMC13042019; doi:10.64898/2026.03.05.709874)
Supplement: 1 [file NIHPP2026.03.05.709874v2-supplement-1.pdf]

bioRxiv preprint doi: https://doi.org/10.64898/2026.03.05.709874; this version posted March 27, 2026. The copyright holder for this preprint (which was not certified by peer review) is the author/funder, who has granted bioRxiv a license to display the preprint in perpetuity. It is made available under aCC-BY 4.0 International license.

Supplemental Materials for

NLP-12/Cholecystokinin signaling stabilizes sensory dendritic structure and protects neuronal healthspan in *Caenorhabditis elegans*

Meera M Krishna[1,2], Swapnil G Waghmare[1,2], Emily C Maccoux[2], Tania Shaik[2], Lezi E[1,2*]

[1]Department of Cell Biology, Neurobiology and Anatomy, Medical College of Wisconsin, 8701 W Watertown Plank Road, Milwaukee, WI 53226, United States

[2]Neuroscience Research Center, Medical College of Wisconsin, 8701 W Watertown Plank Road, Milwaukee, WI 53226, United States

[*]Corresponding author/Lead contact:

lezie@mcw.edu

+1-414-955-2248

bioRxiv preprint doi: https://doi.org/10.64898/2026.03.05.709874; this version posted March 27, 2026. The copyright holder for this preprint (which was not certified by peer review) is the author/funder, who has granted bioRxiv a license to display the preprint in perpetuity. It is made available under aCC-BY 4.0 International license.

Supplemental Figure Legends

Figure S1: Characterization of NLP-12 regulation of PVD aging

a) Lifespan comparison of CT and *nlp-12* mutants (starting *n* = 100). Performed without FUDR. **b-c)** Quantification of number of 4° dendrites *(b)* and menorah *(c)* in CT and *nlp-12* mutants during aging (L4 CT: *n* = 10, L4 *nlp-12* mutant: *n* = 10, D3 CT: *n* = 80, D3 *nlp-12* mutant: *n* = 75, D7 CT: *n* = 82, D7 *nlp-12* mutant: *n* = 53). Dark grey: CT; light pink: *nlp-12(ok335)*. **d-e)** Quantification of number of 6° dendrites normalized to number of 4° branches *(d)* and menorah *(e)* in CT and *nlp-12* mutants during aging (L4 CT: *n* = 10, L4 *nlp-12* mutant: *n* = 10, D3 CT: *n* = 80, D3 *nlp-12* mutant: *n* = 75, D7 CT: *n* = 82, D7 *nlp-12* mutant: *n* = 53). **f)** Comparison of body length in mm between CT and *nlp-12* mutants at D3 (*n* = 14). **g)** Comparison of 6° branches normalized to body size in mm between CT and *nlp-12* mutants at D3 (*n* = 14). **h-j)** Quantification of number of 6˚ branches *(h)*, 4˚ branches *(i)* and menorah *(j)* in CT, *mec-10(tm1552)* and *del-1(ok150)* animals at D3 (*n* = 10 each). **k)** Quantification of number of 6° dendrites in CT (*n* = 89), *nlp-12* mutants (*n* = 79), endogenous promoter driven rescue in *nlp-12* mutant (driven by *nlp-12p*, line 2) (*n* = 89) at D3. **l)** Quantification of number of 6° dendrites in CT (*n* = 50), *nlp-12* mutants (*n* = 56), endogenous promoter driven rescue in *nlp-12* mutant (driven by *nlp-12p*, line 1) (*n* = 58) at D3 and human cholecystokinin (CCK) rescue in *nlp-12* mutant (driven by *nlp-12p* with *nlp-12* signal peptide, line 2) (*n* = 30) at D3. Survival data were analyzed using Kaplan–Meier analysis with log-rank (Mantel–Cox) tests. For two group comparisons, unpaired two-tailed *t*-tests for parametric data or Mann–Whitney tests for non-parametric data were used. For multiple group comparisons Kruskal–Wallis tests with Dunn's correction were used for non-parametric datasets. ns—not significant, * $p < 0.05$, ** $p <0.01$, *** $p < 0.001$, **** $p < 0.0001$.

bioRxiv preprint doi: https://doi.org/10.64898/2026.03.05.709874; this version posted March 27, 2026. The copyright holder for this preprint (which was not certified by peer review) is the author/funder, who has granted bioRxiv a license to display the preprint in perpetuity. It is made available under aCC-BY 4.0 International license.

Figure S2: NLP-12 secretion is important for its role in regulating PVD aging

a) qPCR analysis of *nlp-12* mRNA expression levels at L4 and D7 in CT animals represented as relative quantity, with *act-2* and *cdc-42* as housekeeping genes. **b)** Quantification of number of 6° dendrites in CT (n = 76), *nlp-12* mutants (n = 81), endogenous promoter driven rescue in *nlp-12* mutant (driven by *nlp-12p*, line 1) (n = 86), and mutated signal sequence driven rescue in *nlp-12* mutant (driven by *nlp-12p*, line 2) (n = 60) at D3. Mann–Whitney test was used for two-group comparisons and Kruskal–Wallis test with Dunn's correction was used for multiple group comparison. ns—not significant, **** $p < 0.0001$.

bioRxiv preprint doi: https://doi.org/10.64898/2026.03.05.709874; this version posted March 27, 2026. The copyright holder for this preprint (which was not certified by peer review) is the author/funder, who has granted bioRxiv a license to display the preprint in perpetuity. It is made available under aCC-BY 4.0 International license.

Figure S3: Overexpression of *nlp-12* is neuroprotective

a) Quantification of number of 6º dendrites in CT (*n* = 65) and *nlp-12* OE driven by *nlp-12p* (line 2) (*n* = 63) at D9. **c)** Lifespan comparison of CT and *nlp-12* OE driven by *nlp-12p* (line 1 and line 2) (starting *n* = 100). No statistically significant difference between CT and OE animals unless indicated. Lifespan experiment performed without FUDR. Mann–Whitney test was used for two group comparison and survival data was evaluated using Kaplan–Meier analysis with log-rank (Mantel–Cox) tests. ** $p < 0.01$, **** $p < 0.0001$.

bioRxiv preprint doi: https://doi.org/10.64898/2026.03.05.709874; this version posted March 27, 2026. The copyright holder for this preprint (which was not certified by peer review) is the author/funder, who has granted bioRxiv a license to display the preprint in perpetuity. It is made available under aCC-BY 4.0 International license.

Figure S4: Histamine-based silencing is specific to the DVA neuron

a) Quantification of number of 6° branches in CT (n = 16) and DVA::HisCl1 (*nlp-12p* driven expression of *Drosophila* HisCl1 channel, line 2, n = 23) at D3. **b)** Comparison of pharyngeal pumping in CT (n = 17) and *nlp-12* mutants (n = 8) during a 30 second window at D1. **c)** Quantification of number of 6° branches in CT (untreated n = 24, treated n = 26) and DVA::HisCl1 (line 1, untreated, n = 14) at D3. – indicates absence and + indicates presence of histamine on plates from L4 to D3 stage. Experiments performed without FUDR. Mann-Whitney test was used for two group comparison and Kruskal–Wallis test with Dunn's correction was used for multiple group comparison. ns—not significant, * $p < 0.05$.

bioRxiv preprint doi: https://doi.org/10.64898/2026.03.05.709874; this version posted March 27, 2026. The copyright holder for this preprint (which was not certified by peer review) is the author/funder, who has granted bioRxiv a license to display the preprint in perpetuity. It is made available under aCC-BY 4.0 International license.

Figure S5 Not all GPCRs are NLP-12 receptor candidates

a) Quantification of number of 6˚ branches in CT (*n* = 59), *col-120(sy1526)* (*n* = 60), *nlp-12(ok335)* (*n* = 56) and *col-120(sy1526);nlp-12(ok335)* (*n* = 48) animals at D3. **b)** Quantification of number of 6˚ branches in CT, *nlp-12* OE (driven by *nlp-12p*, line 1), *nlp-12* OE (driven by *nlp-12p*, line 1) in *col-120(sy1526)* background at D9 (*n* = 10 each). **c)** Quantification of number of 6° branches in CT, *npr-2(ok419)* mutants, and *npr-5(ok1583)* mutants at D3 (*n* = 10 each). Mann-Whitney test was used for two group comparison. ns—not significant.

bioRxiv preprint doi: https://doi.org/10.64898/2026.03.05.709874; this version posted March 27, 2026. The copyright holder for this preprint (which was not certified by peer review) is the author/funder, who has granted bioRxiv a license to display the preprint in perpetuity. It is made available under aCC-BY 4.0 International license.

Supplemental Figures

Figure S1

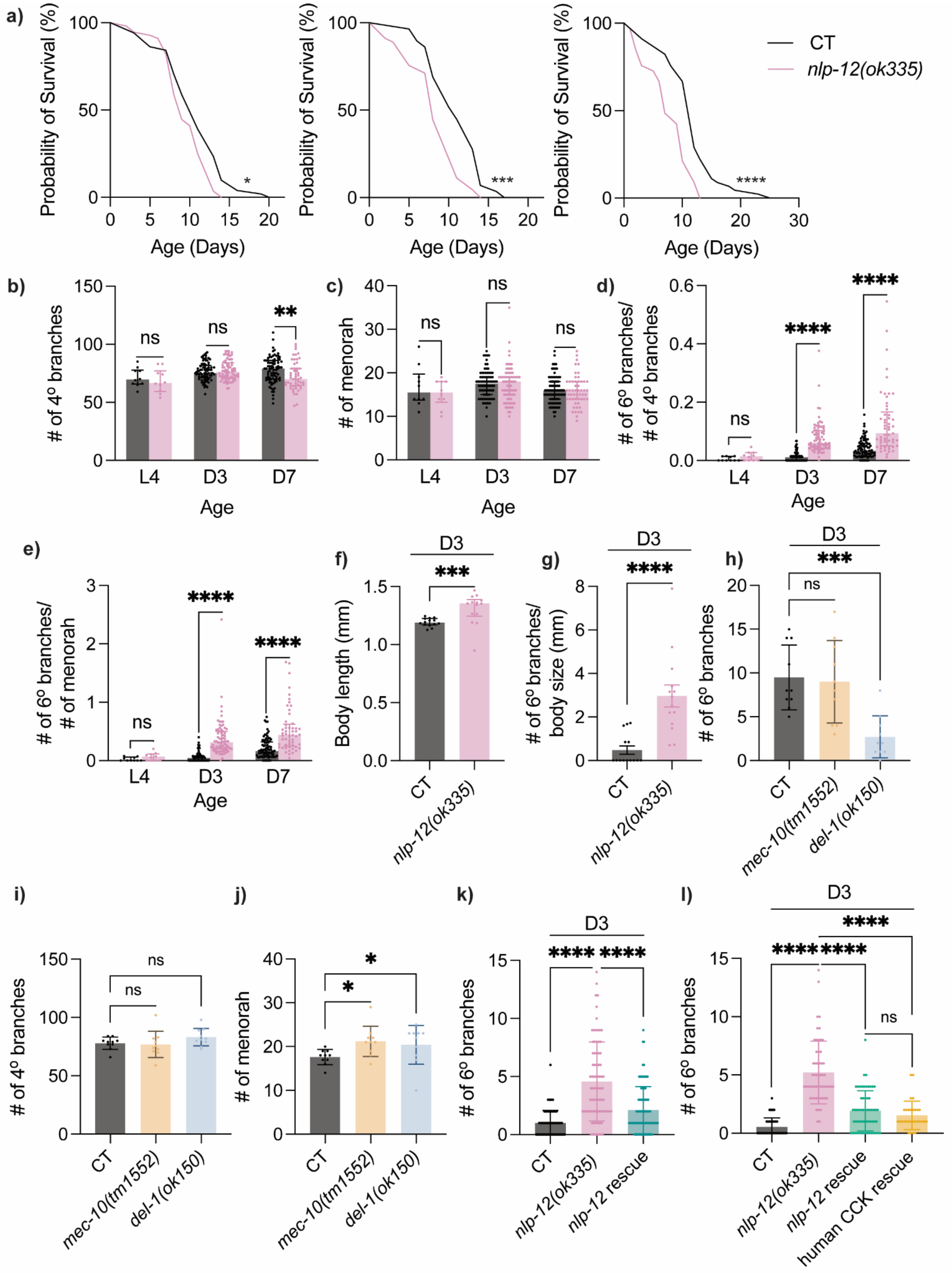

bioRxiv preprint doi: https://doi.org/10.64898/2026.03.05.709874; this version posted March 27, 2026. The copyright holder for this preprint (which was not certified by peer review) is the author/funder, who has granted bioRxiv a license to display the preprint in perpetuity. It is made available under aCC-BY 4.0 International license.

Figure S2

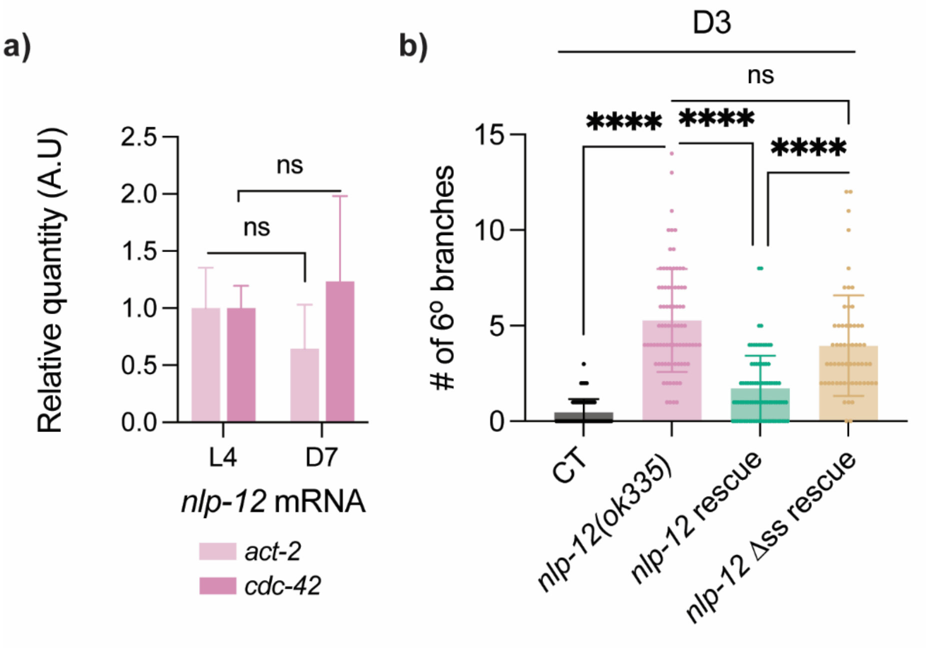

bioRxiv preprint doi: https://doi.org/10.64898/2026.03.05.709874; this version posted March 27, 2026. The copyright holder for this preprint (which was not certified by peer review) is the author/funder, who has granted bioRxiv a license to display the preprint in perpetuity. It is made available under aCC-BY 4.0 International license.

Figure S3

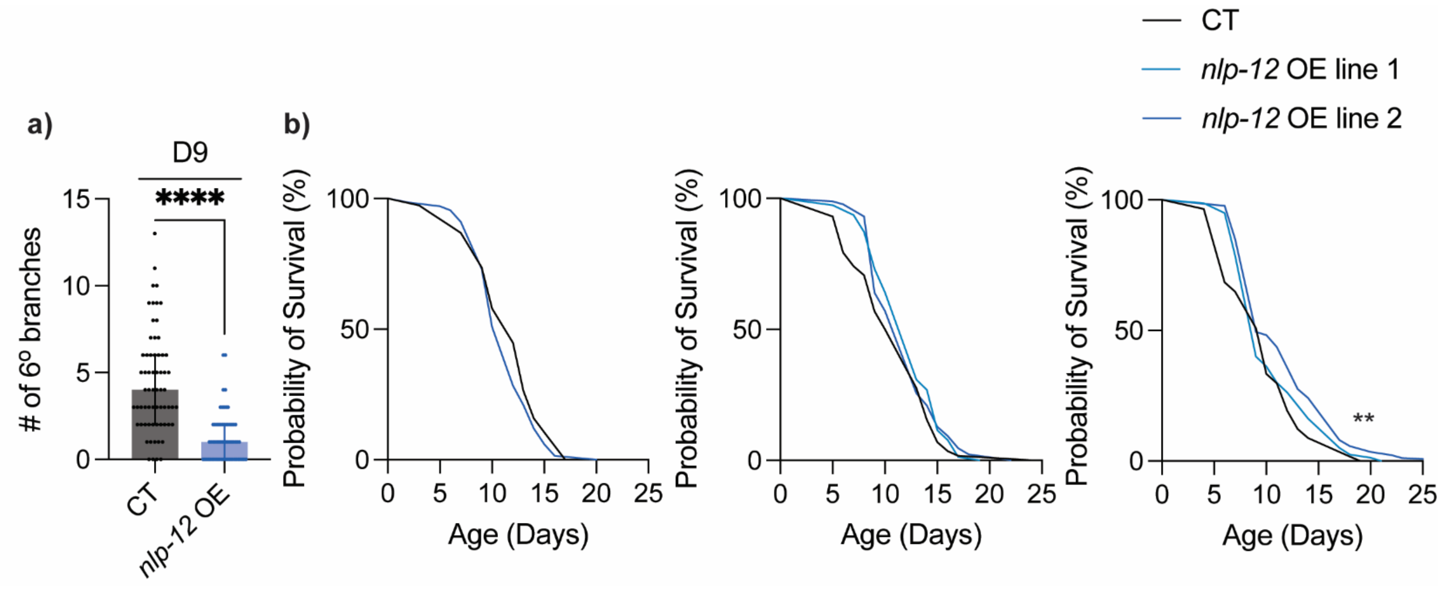

bioRxiv preprint doi: https://doi.org/10.64898/2026.03.05.709874; this version posted March 27, 2026. The copyright holder for this preprint (which was not certified by peer review) is the author/funder, who has granted bioRxiv a license to display the preprint in perpetuity. It is made available under aCC-BY 4.0 International license.

Figure S4

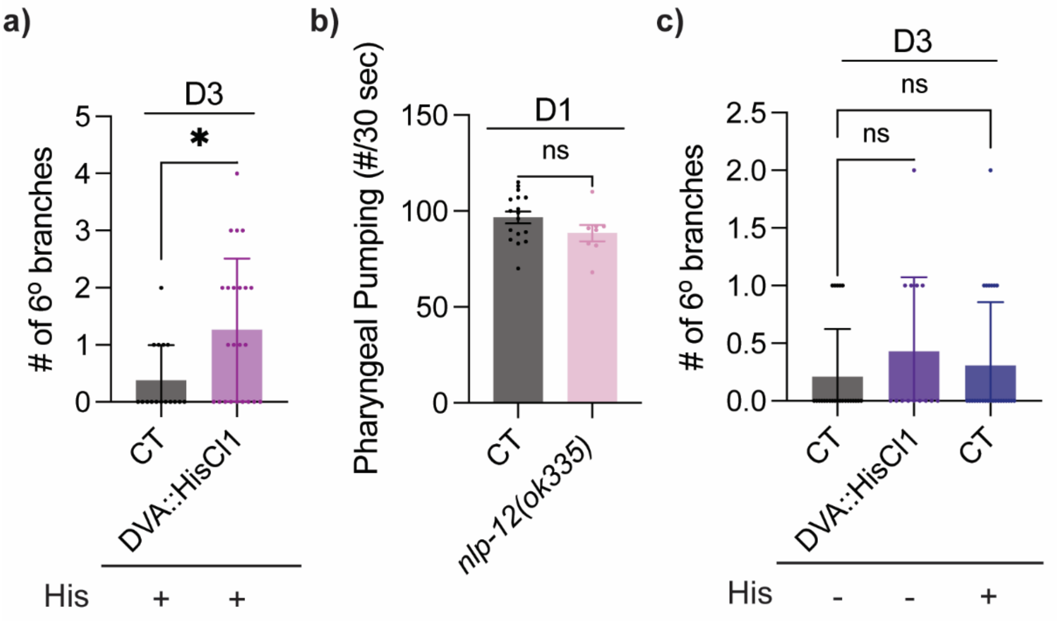

bioRxiv preprint doi: https://doi.org/10.64898/2026.03.05.709874; this version posted March 27, 2026. The copyright holder for this preprint (which was not certified by peer review) is the author/funder, who has granted bioRxiv a license to display the preprint in perpetuity. It is made available under aCC-BY 4.0 International license.

Figure S5

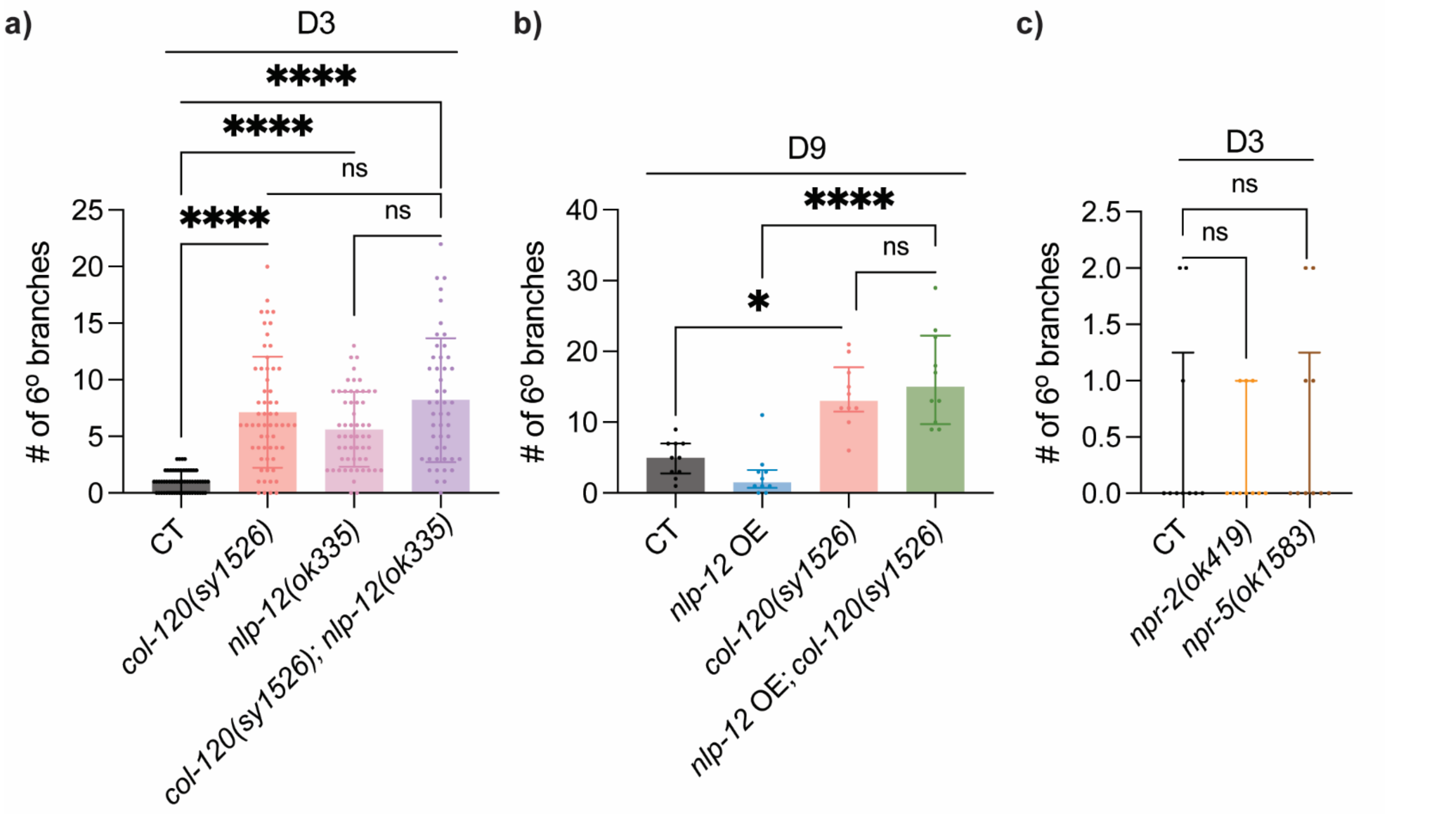

bioRxiv preprint doi: https://doi.org/10.64898/2026.03.05.709874; this version posted March 27, 2026. The copyright holder for this preprint (which was not certified by peer review) is the author/funder, who has granted bioRxiv a license to display the preprint in perpetuity. It is made available under aCC-BY 4.0 International license.

Supplemental Tables

Table S1: List of Strains

Strain	Genotype	Notes
N2	Wild type	CGC
CHS1083	*ckr-1(yum1505)I; ckr-2(yum1506)III*	CGC, used to create ELZ299, ELZ300, ELZ301
CX14394	*npr-5(ok1583)V*	CGC, used to create ELZ295
EG4443	*unc-122p::GFP + unc-119(+)(oxIs253)II; unc-119(ed3) III.*	CGC, used to create ELZ285
ELZ55	*F49H12.4::GFP; unc-119(+)(wdIs51)X*	1x outcross of NC1686
ELZ178	*col-120(sy1526)IV*	3x outcross of PS8819
ELZ250	*ser-2(prom3)::myr::GFP + odr-1p::RFP(wyIs592)III; mec-10(tm1552)X*	ZB2551 crossed with TV15911
ELZ251	*ser-2(prom3)::myr::GFP + odr-1p::RFP(wyIs592)III; del-1(ok150)X*	NC279 crossed with TV15911
ELZ262	*nlp-12(ok335)I; F49H12.4::GFP; unc-119(+)(wdIs51) X*	RB607 crossed with ELZ55
ELZ274	*nlp-12(ok335)I; col-120(sy1526)IV; F49H12,4::GFP;unc-119(+)(wdIs51)X*	ELZ262 crossed with ELZ178
ELZ282	*nlp-12(ok335)I; F49H12,4::GFP;unc-119(+)(wdIs51)X; nlp-12p::nlp-12(gDNA)::mKate(lxyEx128)*	10 ng/µL PELZ140 + 50 ng/µL PELZ4 and 50 ng/µL DNA ladder into ELZ262 (*nlp-12* endogenous rescue line 2)
ELZ283	*col-120(sy1526)IV; F49H12,4::GFP;unc-119(+)(wdIs51)X*	ELZ178 crossed with ELZ55
ELZ285	*unc-122p::GFP + unc-119(+)(oxIs253)II; unc-119(ed3) III; NLP-12::mKate(lxyEx120)*	10 ng/µL PELZ140 + 50 ng/uL PELZ4 injected into ELZ55
ELZ287	*nlp-12(ok335)I; F49H12,4::GFP;unc-119(+)(wdIs51)X; nlp-12p::nlp-12(gDNA)::mKate(lxyEx128)*	10 ng/µL PELZ140 + 50 ng/µL PELZ4 and 50 ng/µL DNA ladder into ELZ262 (*nlp-12* endogenous rescue line 1)
ELZ288	*F49H12,4::GFP;unc-119(+)(wdIs51)X; nlp-12p::nlp-12(gDNA)::nlp-12 3'UTR(lxyEx130)*	30 ng/µL PELZ135 + 50 ng/µL PELZ4 + 50 ng/µL DNA ladder injected into ELZ55 (*nlp-12* overexpression line 2)
ELZ289	*F49H12,4::GFP;unc-119(+)(wdIs51)X; nlp-12p::nlp-12(gDNA)::nlp-12 3'UTR(lxyEx131)*	30 ng/µL PELZ135 + 50 ng/µL PELZ4 + 50 ng/µL DNA ladder injected into ELZ55 (*nlp-12* overexpression line 1)
ELZ292	*col-120(sy1526)IV; F49H12,4::GFP;unc-119(+)(wdIs51)X; nlp-12p::nlp-12(gDNA)::nlp-12 3'UTR(lxyEx131)*	ELZ289 crossed with ELZ178
ELZ294	*npr-2(ok419)IV; F49H12,4::GFP;unc-119(+)(wdIs51)X*	XA3702 crossed with ELZ55
ELZ295	*npr-5(ok1583)V; F49H12,4::GFP;unc-119(+)(wdIs51)X*	CX14394 crossed with ELZ55
ELZ299	*ckr-1(yum1505)I; F49H12,4::GFP;unc-119(+)(wdIs51)X*	CHS1083 crossed with ELZ55 (selected for *yum1505*)

bioRxiv preprint doi: https://doi.org/10.64898/2026.03.05.709874; this version posted March 27, 2026. The copyright holder for this preprint (which was not certified by peer review) is the author/funder, who has granted bioRxiv a license to display the preprint in perpetuity. It is made available under aCC-BY 4.0 International license.

ELZ300	*ckr-2(yum1506)III; F49H12,4::GFP;unc-119(+)(wdIs51)X*	CHS1083 crossed with ELZ55 (selected for *yum1506*)
ELZ301	*ckr-1(yum1505)I; ckr-2(yum1506)III; F49H12,4::GFP;unc-119(+)(wdIs51)X*	CHS1083 crossed with ELZ55
ELZ303	*F49H12,4::GFP;unc-119(+)(wdIs51)X; myo-2p::HisCl::SL2::mScarlet::tbb-2 3'UTR(lxyEx134)*	10 ng /µL pHW1385 + 50 ng/µL PELZ4 + 50 ng/µL DNA ladder injected into ELZ55
ELZ304	*nlp-12(ok335)I; F49H12,4::GFP;unc-119(+)(wdIs51)X; nlp-12p::nlp-12 signal sequence::hCCK::nlp-12 3'UTR(lxyEx135)*	1 ng/µL PELZ151 + 50 ng/µL PELZ4 + 50 ng/µL DNA ladder injected into ELZ262 (hCCK rescue line 2)
ELZ305	*nlp-12(ok335)I; F49H12,4::GFP;unc-119(+)(wdIs51)X; nlp-12p::nlp-12 signal sequence::hCCK::nlp-12 3'UTR(lxyEx136)*	1 ng/µL PELZ151 + 50 ng/µL PELZ4 + 50 ng/µL DNA ladder injected into ELZ262 (hCCK rescue line 1)
ELZ306	*nlp-12(ok335)I; F49H12,4::GFP;unc-119(+)(wdIs51)X; nlp-12p::nlp-12(gDNA mutated signal sequence L9E; L10E; M11E; L12E)::mKate(lxyEx137)*	10 ng/µL PELZ152 + 50 ng/µL PELZ4 + 50 ng/µL DNA ladder injected into ELZ262 (*nlp-12* Δss rescue line 1)
ELZ307	*nlp-12(ok335)I; F49H12,4::GFP;unc-119(+)(wdIs51)X; nlp-12p::nlp-12(gDNA mutated signal sequence L9E; L10E; M11E; L12E)::mKate(lxyEx138)*	10 ng/µL PELZ152 + 50 ng/µL PELZ4 + 50 ng/µL DNA ladder injected into ELZ262 (*nlp-12* Δss rescue line 2)
ELZ308	*ckr-1(yum1505)I; F49H12,4::GFP;unc-119(+)(wdIs51)X; nlp-12p::nlp-12(gDNA)::nlp-12 3'UTR(lxyEx131)*	ELZ289 crossed with ELZ299
ELZ309	*F49H12,4::GFP;unc-119(+)(wdIs51)X; nlp-12p::SI::HisCl::SL2::mScarlet::tbb-2 3'UTR(lxyEx139)*	10 ng/µL PELZ145+ 50 ng/µL PELZ4 + 50 ng/µL DNA ladder injected into ELZ55 (DVA HisCl1 line 2)
ELZ310	*F49H12,4::GFP;unc-119(+)(wdIs51)X; nlp-12p::HisCl::SL2::mScarlet::tbb-2 3'UTR(lxyEx140)*	1 ng/µL PELZ149 + 50 ng/µL PELZ4 + 50 ng/µL DNA ladder injected into ELZ55 (DVA HisCl1 line 1)
NC279	*del-1(ok150)X*	CGC, used to create ELZ251
NC1686	*F49H12.4::GFP; unc-119(+)(wdIs51)X*	CGC, used to create ELZ55
NM4244	*jsIs973 [mec-7p::mRFP + unc-119(+)] III; jsIs609 [mec7p::mtGFP + lin-15(+)] X*	CGC, used to create ELZ237
PS8819	*col-120(sy1526)IV*	CGC, used to create ELZ60
RB607	*nlp-12(ok335)I*	CGC, used to create ELZ262
TV15911	*ser-2(prom3)::myr::GFP + odr-1p::RFP(wyIs592)III*	CGC, used to create ELZ250, ELZ251
XA3702	*npr-2(ok419)IV*	CGC, used to create ELZ294
ZB2551	*mec-10(tm2551)X*	CGC, used to create ELZ250

bioRxiv preprint doi: https://doi.org/10.64898/2026.03.05.709874; this version posted March 27, 2026. The copyright holder for this preprint (which was not certified by peer review) is the author/funder, who has granted bioRxiv a license to display the preprint in perpetuity. It is made available under aCC-BY 4.0 International license.

Table S2: List of Plasmids

Plasmid number	Plasmid description
PC115339	CCK ORF vector (Human) [Applied Biological Materials]
PELZ4	*ttx-3p::GFP*
PELZ5	*ttx-3p::RFP*
PELZ135	*nlp-12p::nlp-12(gDNA)::nlp-12 3'UTR*
PELZ140	*nlp-12p::nlp-12(gDNA)::mKate*
PELZ145	*nlp-12p::SI::HisCl::SL2::mScarlet::tbb-2 3'UTR*
PELZ149	*nlp-12p::HisCl::SL2::mScarlet::tbb-2 3'UTR*
PELZ151	*nlp-12p::nlp-12 signal sequence::hCCK::nlp-12 3'UTR*
PELZ152	*nlp-12p::nlp-12(gDNA mutated signal sequence L9E; L10E; M11E; L12E)::mKate*
pHW1385	*myo-2p::HisCl::SL2::mScarlet::tbb-2 3'UTR* [Addgene]

bioRxiv preprint doi: https://doi.org/10.64898/2026.03.05.709874; this version posted March 27, 2026. The copyright holder for this preprint (which was not certified by peer review) is the author/funder, who has granted bioRxiv a license to display the preprint in perpetuity. It is made available under aCC-BY 4.0 International license.

Table S3: List of Primers

Primer name	Primer sequence (5'-3')	Purpose
act-2 Forward	GGTCGGTATGGGACAGAAAG	qPCR Forward primer for *act-2*
act-2 Reverse	GTGTGATGCCAGATTTTTTC	qPCR Reverse primer for *act-2*
cdc-42 Forward	GTTTGCTTCTCCGTGGTTG	qPCR Forward primer for *cdc-42*
cdc-42 Reverse	GCCAGTTTCTCGAGCATTC	qPCR Reverse primer for *cdc-42*
ckr-1(yum1505) F	CTTAGATGAGATGCTAGCACCTGAGATC	Forward genotyping primer for *ckr-1* (mutants have EcoRI cut site)
ckr-1(yum1505) R	TCGTAAAAGGTTCGGATTTCTAACTGGG	Reverse genotyping primer for *ckr-1* (mutants have EcoRI cut site)
ckr-2(yum1506) F	ACAGTATCTCTTGATGGGGTTGAGTTG	Forward genotyping primer for *ckr-2* (mutants have EcoRI cut site)
ckr-2(yum1506) R	CCCACACAAGTGTGATCATTGCGTAG	Reverse genotyping primer for *ckr-2* (mutants have EcoRI cut site)
col-120(sy2156)-F	GTTGCTTTCGTGGCGACTG	Forward genotyping primer for *col-120*
col-120(sy2156)-R	CCTCCATATCCAGCTTGTC	Reverse genotyping primer for *col-120*
del-1(ok150) F	AAACCAACTGACCCAAGGTG	Forward genotyping primer for *del-1*
del-1(ok150) F2	GCGTAAAAAATTATTCAGACTTCACATTG	Second forward genotyping primer for *del-1*
del-1(ok150)R	TATCTAGGGTCCGCACAACC	Reverse genotyping primer for *del-1*
mec-10(tm1552) F	TGTTTCCGTGCTTATAATAATATCATG	Forward genotyping primer for *mec-10*
mec-10(tm1552) R2	CTGCTCCTGAAAAAAATCTGTTGAC	Second reverse genotyping primer for *mec-10*
mec-10(tm1552) R	AAATACACGGCTCCTTCTTGAG	Reverse genotyping primer for *mec-10*
nlp-12 Forward	TGCTCATGCTCATCCTCGTATTC	qPCR Forward primer for *nlp-12*
nlp-12 Reverse	AATGGACGGTACCCATCTCG	qPCR Reverse primer for *nlp-12*
nlp-12(ok335) F	CACCTCGACTCGGCGAAACTTATTTC	Forward genotyping primer for *nlp-12*
nlp-12(ok335) R2	GGCTGTCCCATTACGGTTTGATCTAC	Second reverse genotyping primer for *nlp-12*
nlp-12(ok335) R	ACCAAAATTTTTGGACAAACC	Reverse genotyping primer for *nlp-12*
nlp-12 F	GCTGGAATTCGCCCTTATTGCATTCTAGA<u>GAGACGAATCCGGAGGATAC</u>	Forward primer for amplifying *nlp-12* gDNA
nlp-12 R	GTGTGCGGTTTTTTCTATGATGTTTAAAAAG<u>CTTTATTCAAAGTTTTTTG</u>	Reverse primer for amplifying *nlp-12* gDNA
